# Unilateral Phrenic Nerve Palsy in Infants with Congenital Zika Syndrome

**DOI:** 10.3201/eid2408.180057

**Published:** 2018-08

**Authors:** Nipunie S. Rajapakse, Kevin Ellsworth, Rachael M. Liesman, Mai Lan Ho, Nancy Henry, Elitza S. Theel, Adam Wallace, Ana Catarina Ishigami Alvino, Luisa Medeiros de Mello, Jucille Meneses

**Affiliations:** Author affiliations: Mayo Clinic, Rochester, Minnesota, USA (N.S. Rajapakse, K. Ellsworth, R.M. Liesman, M.L. Ho, N. Henry, E.S. Theel, A. Wallace);; Professor Fernando Figueira Integral Medicine Institute, Recife, Brazil (A.C. Ishigami Alvino, L.M. de Mello, J. Meneses)

**Keywords:** Zika virus, peripheral nervous system, fetal diseases, phrenic nerve, viruses, congenital Zika syndrome, phrenic nerve palsy

## Abstract

Since the first identification of neonatal microcephaly cases associated with congenital Zika virus infection in Brazil in 2015, a distinctive constellation of clinical features of congenital Zika syndrome has been described. Fetal brain disruption sequence is hypothesized to underlie the devastating effects of the virus on the central nervous system. However, little is known about the effects of congenital Zika virus infection on the peripheral nervous system. We describe a series of 4 cases of right unilateral diaphragmatic paralysis in infants with congenital Zika syndrome suggesting peripheral nervous system involvement and Zika virus as a unique congenital infectious cause of this finding. All the patients described also had arthrogryposis (including talipes equinovarus) and died from complications related to progressive respiratory failure.

## Abstract 2

This case series of right unilateral diaphragmatic paralysis suggests peripheral nervous system involvement.

Zika virus is a neurotropic flavivirus transmitted primarily by *Aedes* spp. mosquitoes (*1**,**2*). Vertical transmission results in congenital infection, the full spectrum of effects of which are yet to be completely defined. The devastating central nervous system (CNS) effects of congenital Zika virus infection have been described in multiple publications since the onset of the recent outbreak in Brazil in 2015 (*3**–**5*). Neural progenitor cells have been identified as a primary viral target in the CNS (*6**,**7*). Defects in neuronal proliferation, migration, and differentiation have also been shown to play a major role in the pathogenesis of congenital Zika syndrome (CZS) (*6**,**7*). Aside from a known association with Guillain-Barré syndrome, the effects of Zika virus infection on the peripheral nervous system (PNS) remains poorly understood, especially in the context of congenital infection (*8*). We describe 4 cases of right unilateral diaphragmatic paralysis in infants with CZS.

## Methods

We obtained clinical, laboratory, and radiologic data from a chart review of patients with diagnoses of CZS who were noted to have unilateral elevation of the diaphragm on chest radiograph. We defined cases of CZS by both the presence of clinical features suggestive of CZS and the detection of Zika virus RNA, Zika virus neutralizing antibodies, or both in serum samples, cerebrospinal fluid (CSF) samples, or both. We tested for Zika virus RNA using the Zika Virus RNA Qualitative Real-Time RT-PCR test (Focus Diagnostics, San Juan Capistrano, CA, USA) on serum and urine in patient 1 and by the CDC Trioplex RT-PCR assay in patients 2–4 (*2*)*.* We screened all patients for Zika virus IgM by using an IgM antibody capture ELISA (MAC-ELISA) that had received US Food and Drug Administration emergency use authorization; we tested samples from patient 1 by the InBios ZIKV Detect MAC-ELISA (InBios International Inc., Seattle, WA, USA) and tested samples from patients 2–4 by the CDC Zika MAC-ELISA (*9**,**10*)*.* Following US CDC recommendations, all specimens that screened positive for IgM to Zika virus were confirmed by PRNT (Table). We classified the HC of all patients according to the International Fetal and Newborn Growth Consortium (INTERGROWTH-21st) to determine z-scores (*11*). We defined severe microcephaly as HC z-score <–3 for sex and gestational age. We defined arthrogryposis as >2 joint contractures involving the upper and/or lower limbs.

**Table:** Summary of maternal and infant characteristics in 4 cases of congenital Zika syndrome in infants who had unilateral elevation of the diaphragm*

Characteristics	Patient 1	Patient 2	Patient 3	Patient 4
Maternal characteristics				
Maternal age, y	27	21	17	21
Prenatal care	Yes	Yes	Yes	Yes
Zika virus symptoms, trimester	1st	1st	1st	1st
Fever	+	–	+	–
Rash	+	+	–	+
Arthralgia	+	–	–	–
Abnormal fetal ultrasound, trimester	3rd	3rd	2nd	3rd
Infant characteristics				
Delivery type	Cesarean	Cesarean	Vaginal	Cesarean
Sex	F	F	F	F
Gestational age, wk	38	40	39	41
Birthweight, g	2,020	2,025	2,565	2,075
Length, cm	40	42	46	41
HC at birth, cm	27.5	28.5	28	28
z-score	−4.3	−4.3	−4.2	−4.9
Arthrogryposis	Hips, knees, ankles, elbows	Hips, ankles, wrists	Hips, ankles, wrists	Hips, ankles, wrists
Talipes equinovarus	+	+	+	+
Head imaging findings	V, C, H	V	V, C, H	V, C
Elevated right hemidiaphragm	+	+	+	+
Cause of death	Respiratory failure	Respiratory failure	Respiratory failure	Respiratory failure
Day of life	13	10	86	4
Maternal testing for Zika virus				
RT-PCR				
Amniotic fluid	NA	NA	+	NA
Serum	NA	NA	–	–
IgM, serum	–	NA	–	–
Infant testing				
RT-PCR for Zika virus				
Serum	–	–	–	–
CSF	NA	–	–	+
Urine	–	NA	NA	NA
Placenta	–	NA	NA	NA
Zika virus IgM				
Serum	+	+	+	+
CSF	NA	+	+	+
PRNT titer				
Zika virus	>1:1,280	180†	897†	270†
DENV-1	1:10	<20	<20	<20
DENV-2	<1:10	<20	<20	<20

*C, calcifications; CSF, cerebrospinal fluid; DENV, dengue virus; H, cerebral hypoplasia; HC, head circumference; NA, not available; PRNT, plaque reduction neutralization test; RT-PCR, reverse transcription PCR; V, ventriculomegaly; +, positive finding/test result; –, negative finding/test result. †Indicates 50% plaque reduction neutralization test titer.

## Cases

### Patient 1

A female infant was delivered at 38 weeks’ gestation via cesarean section for breech presentation to a 27-year-old previously healthy primigravida woman. The mother reported having an illness characterized by mild fever, myalgia, arthralgia, and a generalized pruritic maculopapular rash at 4–6 weeks’ gestation, while the mother was living in rural Guatemala (Table). The illness lasted 5–7 days and was self-limited. The mother did not seek medical attention during that time, and no diagnostic testing was performed.

At 30 weeks’ gestation, the mother immigrated to the United States. Fetal ultrasound at 34 weeks’ gestation revealed severe fetal growth restriction (below the second percentile), severe microcephaly (>5 SD below the norm, estimated to be 12 weeks delayed), and diffuse intracranial calcifications. The mother declined amniocentesis; noninvasive prenatal screening for aneuploidy was negative. Maternal serologic testing was negative for dengue virus (DENV) and Zika virus IgM and negative for evidence of recent infection with cytomegalovirus, rubella, varicella-zoster virus, syphilis, *Toxoplasma gondii*, HIV, and parvovirus B19.

The infant had a birthweight of 2,020 g and a head circumference of 27.5 cm (z-score −4.3), in keeping with severe microcephaly. Arthrogryposis involving the hips, knees, ankles, and elbows was observed, along with bilateral talipes equinovarus. The infant was intubated shortly after birth because of poor respiratory effort. A computed tomography (CT) scan of the head showed severe microcephaly with intracranial volume loss, including thinning of the cortical mantle, and callosal and pontocerebellar hypoplasia with ex vacuo ventriculomegaly. Multiple dystrophic bandlike calcifications were seen along the corticomedullary junction and periventricular white matter, as well as within the basal ganglia and brainstem. Optic nerves were diminutive. Overriding cranial sutures, prominent occipital shelf, and scalp rugae were confirmatory for fetal brain disruption sequence. Chest radiography revealed a markedly and persistently elevated right hemidiaphragm (Figure, panel A). Molecular testing for Zika virus RNA on infant serum and urine specimens obtained shortly after birth were negative. Lumbar puncture could not be performed safely. Serum Zika virus IgM was positive. Plaque reduction neutralization testing (PRNT) was positive for both Zika virus (titer ≥1:1,280) and DENV-1 (at the cutoff titer of 1:10) but negative for DENV-2. Molecular testing of multiple formalin-fixed paraffin-embedded placental tissue samples for Zika virus RNA was performed by the US Centers for Disease Control and Prevention (CDC), and results were negative. The infant was extubated on day of life 2 and died from progressive respiratory failure on day of life 13. The family declined autopsy.

**Figure:**
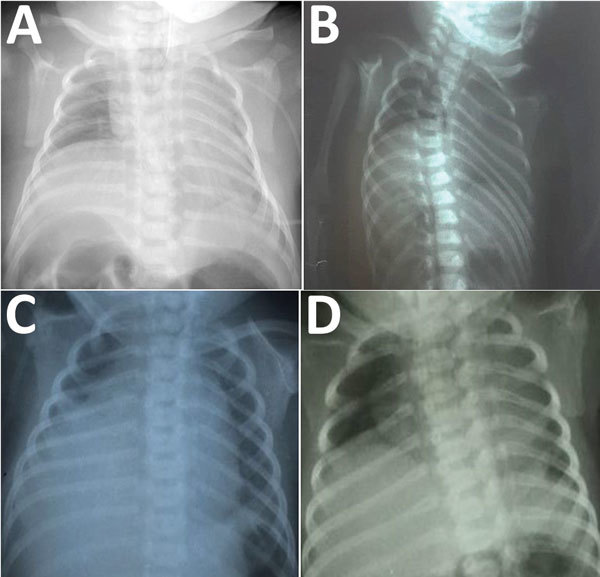
Chest radiographs of infants with congenital Zika syndrome, demonstrating elevation of the right hemidiaphragm. Panels A–D represent patients 1–4, respectively. In each instance, only the right hemidiaphragm was noticeably elevated. All patients also had arthrogryposis (including talipes equinovarus) and died from complications related to progressive respiratory failure.

### Patient 2

A female infant was born at 40 weeks’ gestation to a 21-year-old primigravida mother from Brazil via cesarean section for breech presentation. The mother reported having an illness characterized by rash but no fever during the first trimester of pregnancy (Table). Fetal ultrasound performed during the third trimester indicated microcephaly, ventriculomegaly, cerebral hypoplasia, and intracranial calcifications. Amniocentesis and maternal serologic testing were not performed. At delivery, the infant had a birthweight of 2,025 g and head circumference of 28.5 cm (z-score −4.3) and was noted to have arthrogryposis involving the hips, ankles, and wrists, as well as bilateral talipes equinovarus. Chest radiograph revealed a persistently elevated right hemidiaphragm (Figure, panel B); a head ultrasound confirmed the prior fetal intracranial findings. Infant serum and CSF samples were positive for Zika virus IgM (Table), but Zika virus RNA was not detected in serum or CSF samples. The infant died from respiratory failure on day of life 10.

### Patient 3

A female infant was born at 39 weeks’ gestation to a 17-year-old primigravida mother from Brazil via spontaneous vaginal delivery (*12*). The mother reported having a febrile illness without rash during the first trimester of pregnancy (Table). Fetal ultrasound performed during the second trimester showed microcephaly, ventriculomegaly, cerebral hypoplasia, and intracranial calcifications. An amniocentesis was performed at 29 weeks’ gestation, and Zika virus RNA was detected in the amniotic fluid. The infant’s birthweight was 2,565 g, and head circumference at birth was 28.2 cm (z-score −4.2). The neonate was noted to have arthrogryposis involving the hips, wrists, and ankles, as well as bilateral talipes equinovarus. A head ultrasound confirmed the prior fetal intracranial findings, and chest radiograph revealed an elevated right hemidiaphragm (Figure, panel C). Zika virus IgM was detected in infant serum and CSF samples (Table), but Zika virus RNA was not detected in serum or CSF samples. The infant died from respiratory failure on day of life 86.

### Patient 4

A female infant was born at 41 weeks’ gestation to a 21-year-old primigravida mother from Brazil via cesarean section for breech presentation. The mother reported having an afebrile illness with rash during the first trimester of pregnancy (Table). Fetal ultrasound performed during the third trimester showed microcephaly, ventriculomegaly, and intracranial calcifications. No amniocentesis or maternal serologic testing was performed. The infant had a birthweight of 2,075 g and head circumference of 28 cm (z-score −4.9); arthrogryposis involving the hips, wrists, and ankles; and bilateral talipes equinovarus. Chest radiograph revealed an elevated right hemidiaphragm (Figure, panel D), and the infant required noninvasive respiratory support. Zika virus IgM as detected in both serum and CSF samples (Table). Molecular testing for Zika virus RNA on a serum sample was negative, but Zika virus RNA was detected in a CSF sample. No head imaging could be performed before the infant’s death from respiratory failure on day of life 4.

## Discussion

We report a series of 4 patients with CZS and right unilateral diaphragmatic paralysis suggesting PNS involvement in CZS and Zika virus as a unique congenital infectious cause of this finding. All the patients were female infants born at term to primiparous mothers who reported symptoms suggestive of Zika virus infection during the first trimester of pregnancy. All infants in this case series had severe microcephaly and arthrogryposis and died from progressive respiratory failure.

A recent study of Zika virus–infected macaques reported evidence of viral tropism to the peripheral nerves (*13*). Cases of acute Zika virus infection associated with peripheral sensory neuropathy in a child and an adult also have been published (*14**,**15*). Guillain-Barré syndrome, which is typically a postinfectious phenomenon, is known to involve the phrenic nerve, causing diaphragmatic paralysis in adults, but has not been described in neonates (*8*). Unilateral diaphragmatic paralysis in adults resulting from presumed postviral phrenic neuropathy has been described after infection with varicella zoster virus, poliovirus, West Nile virus, HIV, and DENV but has not been described in newborns or in association with any known congenital infections (*16*–*21*).

Arthrogryposis observed in CZS is thought to be of neurogenic origin, with involvement of both upper and lower motor neurons, resulting in restricted fetal movement and the consequent development of joint contractures (*22*). A recent case series demonstrated thinning of the entire spinal cord and brainstem hypoplasia on magnetic resonance imaging in infants with CZS and arthrogryposis (*23*). These findings were also associated with more severely reduced conus medullaris anterior roots and more frequent periventricular calcifications when compared with infants with CZS without arthrogryposis (*23*). Recent postmortem examinations of 2 neonates with CZS and arthrogryposis also established the presence of Zika virus and associated tissue injury in the spinal cord (*24*). Taken collectively, these results suggest that infants with CZS and arthrogryposis may represent a more severely affected subgroup reflecting earlier fetal infection and possibly more severe interruption in neuronal migration, cortical organization, or both.

Our results indicated that congenital Zika virus infection appears to be an infectious cause of congenital unilateral diaphragmatic paralysis (*9**,**12**,**25*). All patients in this series had arthrogryposis and severe microcephaly, suggesting an association of unilateral diaphragmatic paralysis with severe manifestations of CZS. The precise mechanism(s) by which diaphragmatic paralysis occurs, and why it is consistently unilateral and right-sided in these cases, remains unknown. Potential mechanisms include abnormal diaphragmatic innervation secondary to early interruption of neuronal migration; direct, viral-mediated phrenic nerve or spinal cord injury; or, less likely, a demyelinating neuropathy or other immune-mediated process.

Respiratory insufficiency and subsequent failure have not been commonly reported in CZS; the prominence of respiratory difficulties in the cases presented here is likely secondary to impaired diaphragmatic function. Unilateral diaphragmatic paralysis may represent a unique risk factor for death in infants with CZS, considering that all patients in this case series died within the first 3 months of life (3 of 4 within the first 2 weeks of life). In a previous report of a large cohort (n = 87) of neonates with CZS, only infants with unilateral diaphragmatic paralysis (n = 3; patients 2–4 in this report) died before hospital discharge, and all required respiratory support following birth (*9*). 

The long-term prognosis of infants with CZS and persistent diaphragmatic paralysis who survive the immediate postnatal period is unknown. The true prevalence of this finding is also unknown, because chest radiography has not been routinely recommended for all infants with CZS (*26**,**27*). However, recent guidelines recommend considering diaphragmatic paralysis in infants with CZS who develop respiratory distress or failure or are unable to be weaned from ventilator support (*27*).

Limitations of this study include that the chest radiograph findings could not be confirmed by dynamic imaging studies of the diaphragm (e.g., ultrasound or fluoroscopy) because of patient instability and the risks associated with patient transport to a radiology suite in all the cases presented. Electrodiagnostic studies (electromyography/nerve conduction studies) and nerve biopsies to further investigate the underlying mechanism of the diaphragmatic paralysis also could not be obtained, for similar reasons. The differential diagnosis of right unilateral hemidiaphragm elevation includes diaphragmatic eventration, a hepatic mass, or decreased lung volume (pulmonary hypoplasia, atelectasis); however, there was no evidence of these abnormalities on examination or other imaging studies and, aside from some reports of pulmonary hypoplasia, they have not been previously reported in patients with CZS (*28**,**29*). Bilateral diaphragmatic involvement is also a possibility but may not be obvious on radiographic studies given the presence of the cardiac silhouette on the left side. Autopsies could not be completed in any of the cases presented to exclude these possibilities because of the lack of parental consent.

This case series of infants with CZS, arthrogryposis, and unilateral diaphragmatic paralysis describes a unique constellation of clinical findings that has not been described with other congenital infections. The presence of both arthrogryposis and unilateral diaphragmatic paralysis suggests likely involvement of the PNS, spinal cord, or both in these severely affected infants. The presence of diaphragmatic paralysis may also represent a risk factor for early death in infants born with CZS. Further studies to elucidate the precise mechanism leading to diaphragmatic paralysis in these patients are required.
